# Reduced Systolic Function and Not Genetic Variants Determine Outcome in Pediatric and Adult Left Ventricular Noncompaction Cardiomyopathy

**DOI:** 10.3389/fped.2021.722926

**Published:** 2021-09-03

**Authors:** Alina Schultze-Berndt, Jirko Kühnisch, Christopher Herbst, Franziska Seidel, Nadya Al-Wakeel-Marquard, Josephine Dartsch, Simon Theisen, Walter Knirsch, Rolf Jenni, Matthias Greutmann, Erwin Oechslin, Felix Berger, Sabine Klaassen

**Affiliations:** ^1^Department of Pediatric Cardiology, Charité - Universitätsmedizin Berlin, Corporate Member of Freie Universität Berlin, Humboldt-Universität zu Berlin, Berlin Institute of Health, Berlin, Germany; ^2^Experimental and Clinical Research Center, A Cooperation between the Max-Delbrück-Center for Molecular Medicine in the Helmholtz Association and the Charité - Universitätsmedizin Berlin, Berlin, Germany; ^3^DZHK (German Centre for Cardiovascular Research), Berlin, Germany; ^4^Department of Congenital Heart Disease - Pediatric Cardiology, German Heart Center Berlin, Berlin, Germany; ^5^Institute for Imaging Science and Computational Modelling in Cardiovascular Medicine, Charité - Universitätsmedizin Berlin, Corporate Member of Freie Universität Berlin, Humboldt-Universität zu Berlin, Berlin Institute of Health, Berlin, Germany; ^6^Pediatric Cardiology, Pediatric Heart Center, Department of Surgery, and Children's Research Center, University Children's Hospital Zurich, University of Zurich, Zurich, Switzerland; ^7^University of Zurich, Zurich, Switzerland; ^8^Department of Cardiology, University Heart Center, University of Zurich, Zurich, Switzerland; ^9^Toronto Adult Congenital Heart Disease Program, University Health Network/Toronto General Hospital, Peter Munk Cardiac Centre, Toronto, ON, Canada; ^10^University of Toronto, Toronto, ON, Canada

**Keywords:** cardiomyopathy, pediatric and congenital heart disease, genetics, noncompaction, pediatrics - children

## Abstract

**Background:** Left ventricular noncompaction cardiomyopathy (LVNC CMP) is a genetic cardiomyopathy. Genotype-phenotype correlation and clinical outcome of genetic variants in pediatric and adult LVNC CMP patients are still unclear.

**Methods:** The retrospective multicenter study was conducted in unrelated index patients with LVNC CMP, diagnosed between the years 1987 and 2017, and all available family members. All index patients underwent next-generation sequencing for genetic variants in 174 target genes using the Illumina TruSight Cardio Sequencing Panel. Major adverse cardiac events (MACE) included mechanical circulatory support, heart transplantation, survivor of cardiac death, and/or all-cause death as combined endpoint.

**Results:** Study population included 149 LVNC CMP patients with a median age of 27.8 (9.2–44.8) years at diagnosis; 58% of them were symptomatic, 18% suffered from non-sustained and sustained arrhythmias, and 17% had an implantable cardioverter defibrillator (ICD) implanted. 55/137 patients (40%) were ≤ 18 years at diagnosis.

A total of 134 variants were identified in 87/113 (77%) index patients. 93 variants were classified as variant of unknown significance (VUS), 24 as likely pathogenic and 15 as pathogenic. The genetic yield of (likely) pathogenic variants was 35/113 (31%) index patients. Variants occurred most frequently in *MYH7* (n=19), *TTN* (*n* = 10) and *MYBPC3* (*n* = 8). Altogether, sarcomere gene variants constituted 42.5% (*n* = 57) of all variants. The presence or absence of (likely) pathogenic variants or variants in specific genes did not allow risk stratification for MACE.

Reduced left ventricular (LV) systolic function and increased left ventricular end-diastolic diameter (LVEDD) were risk factors for event-free survival in the Kaplan-Meier analysis. Through multivariate analysis we identified reduced LV systolic function as the main risk factor for MACE. Patients with reduced LV systolic function were at a 4.6-fold higher risk for MACE.

**Conclusions:** Genetic variants did not predict the risk of developing a MACE, neither in the pediatric nor in the adult cohort. Multivariate analysis emphasized reduced LV systolic function as the main independent factor that is elevating the risk for MACE. Genetic screening is useful for cascade screening to identify family members at risk for developing LVNC CMP.

## Introduction

Left ventricular noncompaction cardiomyopathy (LVNC CMP) is a rare genetic cardiomyopathy. LVNC is characterized by prominent trabeculations and deep intertrabecular recesses communicating with the left ventricular cavity; a two-layered myocardium with an at least twice as thick non-compacted than the thinned compacted layer are mandatory phenotypic characteristics (1). LVNC CMP is diagnosed in all age groups (2–4). In children, LVNC CMP is reported to make up around 5–10 % of cardiomyopathies (5, 6). For adults, an incidence of 0.05% was described (7) and the five-year survival was reported to be around 86% (8). LVNC CMP is a very heterogenous clinical disease ranging from asymptomatic to severely affected patients with the need for heart transplantation (Htx) or the risk for sudden cardiac death. Typical symptoms and complications are congestive heart failure, arterial thrombembolism, arrhythmias, and sudden cardiac death (9–11). The diagnosis is mostly made by routine transthoracic 2D Doppler echocardiography and cardiac magnetic resonance (CMR) imaging. Currently, it is difficult to predict the clinical course of the disease. Due to the clinical heterogeneity, it is important to identify high risk patients at an early stage.

Approximately in 50% of patients LVNC has a genetic cause (4). It has been known for a while that sarcomere genes are affected most frequently with around 63% of relevant variants identified (12, 13). A large part of the genetic variants found were also associated with other cardiomyopathies, such as dilated cardiomyopathy (DCM) and hypertrophic cardiomyopathy (HCM) (14, 15). A recent study reported LVNC specific truncating variants in *MYH7, ACTN2* and *PRDM16* (16). Current guidelines recommend genetic testing, although the specific therapeutic implications of the results remain mostly unknown (17).

Van Waning et al. divided the LVNC phenotype into 3 groups, differentiating isolated LVNC CMP from LVNC with DCM and LVNC with HCM (18). It remains unclear whether patients, who phenotypically belong to one of these groups can expect a similar course of disease as patients with DCM or HCM without LVNC. So far, the general incidence of adverse events in adults with LVNC CMP was described being similar to DCM without LVNC, with a slightly higher heart failure admission rate (19). Furthermore, the question remains whether and to what extent the different subtypes of LVNC correlate with genetic equivalents.

In this study, we examined genetics, clinical phenotype and outcome of 113 pediatric and adult index patients with LVNC CMP and their family members. We analyzed retrospective data to compare risk factors for adult and pediatric patients and different subtypes of LVNC CMP to further classify patients for more individual risk stratification and individual therapeutic regimes.

## Methods

### Study Population

The retrospective study consisted of unrelated index patients with LVNC CMP diagnosed between the years 1987 and 2017. Additionally, we included all available affected and unaffected family members. Clinical data was collected through medical records from Charité-Universitätsmedizin Berlin and German Heart Center Berlin, Germany and University Hospital Zurich and University Children's Hospital Zurich, Switzerland. The study was approved by the institutional ethics committees in accordance with the Declaration of Helsinki. All participants and legal guardians of participants under 18 years gave written informed consent.

### Genetic Testing

All index patients underwent next-generation sequencing (NGS) for genetic variants in 174 target genes using the Illumina TruSight Cardio Sequencing Panel. Eighty-nine cardiomyopathy genes were bioinformatically filtered as previously published (20) with a minor allele frequency (MAF) of < 0.0001 (gnomAD reference database, *https://gnomad.broadinstitute.org/)*. Variants were classified according to the guidelines of the American College of Medical Genetics and Genomics (21). Unaffected and affected family members underwent Sanger Sequencing for the variants identified in the respective index patients. These variants included variants classified as (likely) pathogenic and variants of uncertain significance (VUS). The 89 genes which were bioinformatically evaluated were sorted into functional groups as previously published by Kühnisch et al. (20). The index patients were classified into four groups according to the presence of genetic variants: a) patients with no variants; b) patients with only VUS variants; c) patients with only (likely) pathogenic variants; and d) patients with VUS and (likely) pathogenic variants.

### Diagnostic Criteria

LVNC was diagnosed by echocardiography according to the gold standard by Jenni et al. (1). The patients were classified into three phenotypic subtypes: Isolated LVNC CMP, dilated LVNC CMP and hypertrophic LVNC CMP (18). For adult patients, dilated LVNC CMP was diagnosed in patients with an increased left ventricular end-diastolic diameter (LVEDD) ≥54 mm in females and ≥60 mm in males (22). Hypertrophic LVNC CMP was defined by a left ventricular (LV) wall-thickness ≥13 mm (23). For pediatric patients, we used LVEDD and LV wall-thickness >2 standard deviations different from a normal population (24). When both, increased LVEDD and increased LV wall-thickness were found at the same time, we classified the patient as hypertrophic LVNC CMP. Patients with neither increased LVEDD nor increased LV wall thickness were categorized as isolated LVNC CMP. When the values for LVEDD or wall thickness were not available, the patients were excluded from the subtype analysis. Reduced LV systolic function was defined as LV ejection fraction (LV-EF) <45% or fractional shortening <19% in males and <21% in females (22).

**Follow-up**. Follow-up for occurrence of major adverse events (MACE) started with the date of diagnosis including mechanical circulatory support (MCS), HTx, survival of sudden cardiac death, and/or all-cause death as a combined endpoint. Event-free survival was defined as the time to MACE. When dyspnoe, syncope, shock, or palpitations were recorded patients were classified as symptomatic. Arrhythmias included atrioventricular block II°/III°, non-sustained and sustained supraventricular tachycardia, non-sustained and sustained ventricular tachycardia, and atrial fibrillation recorded by 12-lead ECG or Holter-ECG. Body surface area (BSA) was calculated using the Mosteller method (25).

### Statistical Analysis

Statistical analysis was performed using SPSS v.26 (IBM Corporation). For categorical data we used the Pearson x^2^ test. For tables with an expected cell frequency <5, the Fisher exact test was used. Continuous data was compared with the Mann-Whitney U test for 2 independent groups and the Kruskal-Wallis test for >2 independent groups. Odd ratios were calculated using binary logistic regression. For Hazard ratios (HR) we performed Cox regression analysis. Kaplan-Meier curves were used for event-free survival analysis with the time of diagnosis as time point zero. The survival times of different groups were compared with the log-rank test. In the survival analysis, patients were considered at risk until the time of last follow-up, at which they were censored.

## Results

### Clinical Characteristics of LVNC Patients

**Figure 1:**
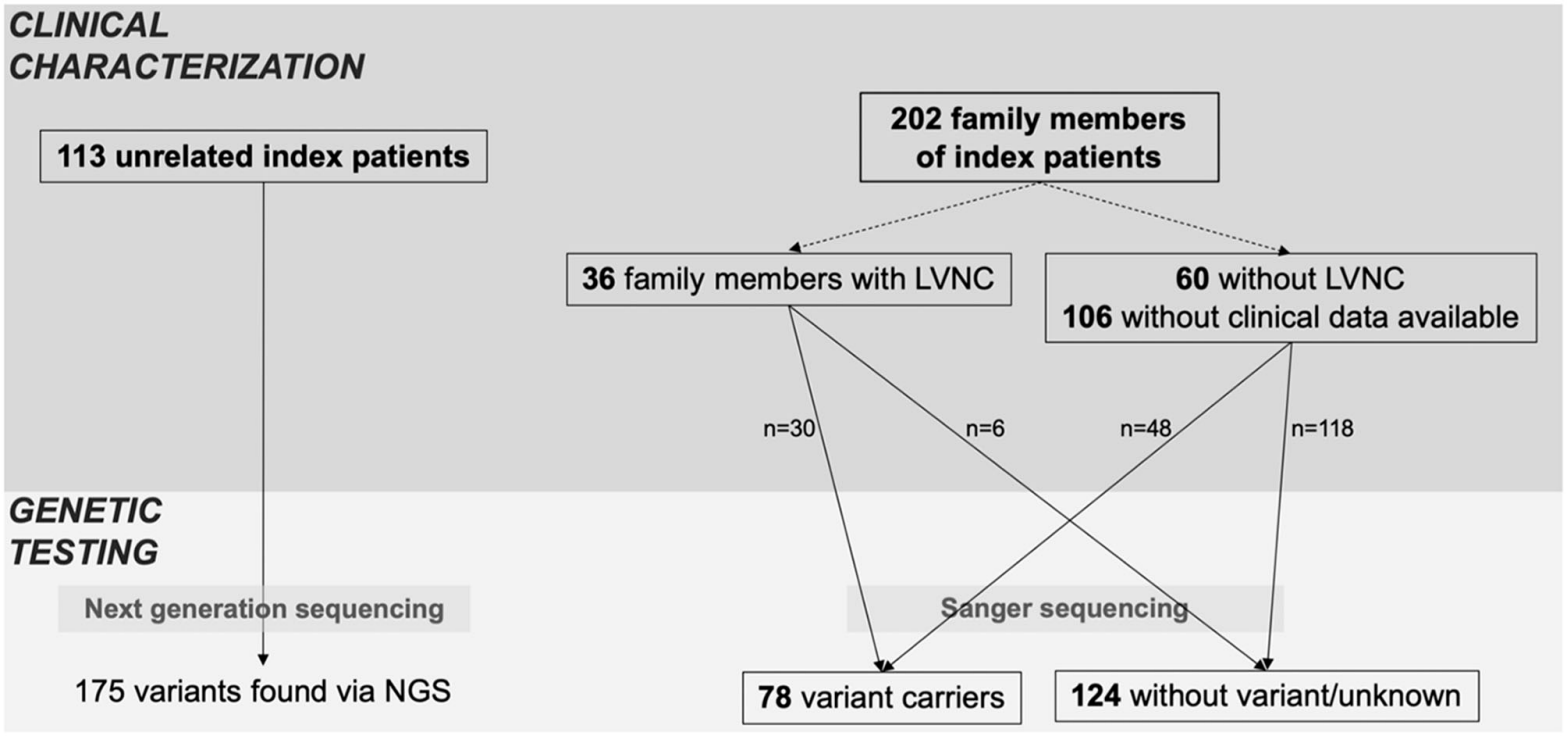
Index patients and family screening. Overview of clinical characterization and genetic testing of patients, including all available family members. Family members were classified as variant carriers, when they carried at least one of the variants of the respective index patient.

As shown in Figure 1, the cohort consisted of 113 unrelated LVNC CMP patients and 202 family members from individual 54 families. Clinical data were available for 96 family members, of which 36 (37.5%) had a diagnosis of CMP and 9 (9.4%) had a hypertrabeculated myocardium without LVNC. Overall, 149 individuals with LVNC CMP were enrolled in the study at a median age of 27.8 (9.2–44.8) years. Of these 149 individuals with LVNC, 58% were symptomatic, 18% suffered from arrhythmias and 17% had an implantable cardioverter defibrillator (ICD) implanted (Table 1). Ventricular tachycardia occurred in 19/149 patients (13%). 55/137 patients (40%) were ≤ 18 years at diagnosis. Ventricular septal defect was the most common congenital heart defect in 12/149 patients (8%), and patent foramen ovale, Ebstein anomaly, patent ductus arteriosus, and other congenital heart defects were also noted (Table 1).

**Table 1:** Clinical characteristics of LVNC patients.

	**All *n* = 149**
Female	61 (41)
Age at diagnosis (yrs)	27.8 (9.2–44.8)
<18 years at diagnosis	55 (40)
Body surface area (m^2^)	1.66 (1.21–1.90)
Symptomatic	76 (58)
**Congenital heart defect**	26 (17)
Ventricular septal defect	12 (8)
Patent foramen ovale	11 (7)
Ebstein anomaly	5 (3)
Patent ductus arteriosus	5 (3)
Other congenital heart defects	5 (3)
**Echocardiography**	
Reduced LV systolic function	65 (46)
LV-EF (%)	47.6 (33.0–62.5)
Increased LVEDD	55 (37)
LVEDD (mm)(patients >18 yrs only)	54.0 (49.0–65.0)
LVEDD (Z-score)(patients <18 yrs only)	1.66 (0.40–4.39)
Increased LVEDD and reduced LVsystolic function	39 (26)
**Subtypes**	
Isolated LVNC	52 (48)
Dilated LVNC	35 (32)
Hypertrophic LVNC	22 (20)
**ECG**	
ST-Depression	20 (13)
T-Inversion	22 (15)
Bundle branch block	22 (15)
**Arrhythmias**	27 (18)
Atrial fibrillation	2 (1)
Atrioventricular block II°/III°	1 (1)
Supraventricular tachycardia	8 (5)
Ventricular tachycardia	19 (13)
ICD	26 (17)
**Follow-up (yrs)**	5.6 (1.7–11.4)
**Complications**	
MACE	27 (18)
HTx	14 (9)
Death	11 (7)

*Values are given as n (%) or median (interquartile range). HTx, Heart transplantation, ICD, Implantable cardioverter defibrillator, LVEDD, Left ventricular end-diastolic diameter, LV, Left ventricular, LVNC, Left ventricular noncompaction cardiomyopathy, LV-EF, Left ventricular ejection fraction, MACE, Major adverse cardiac events*.

### Genetic Findings in Index Patients

**Figure 2:**
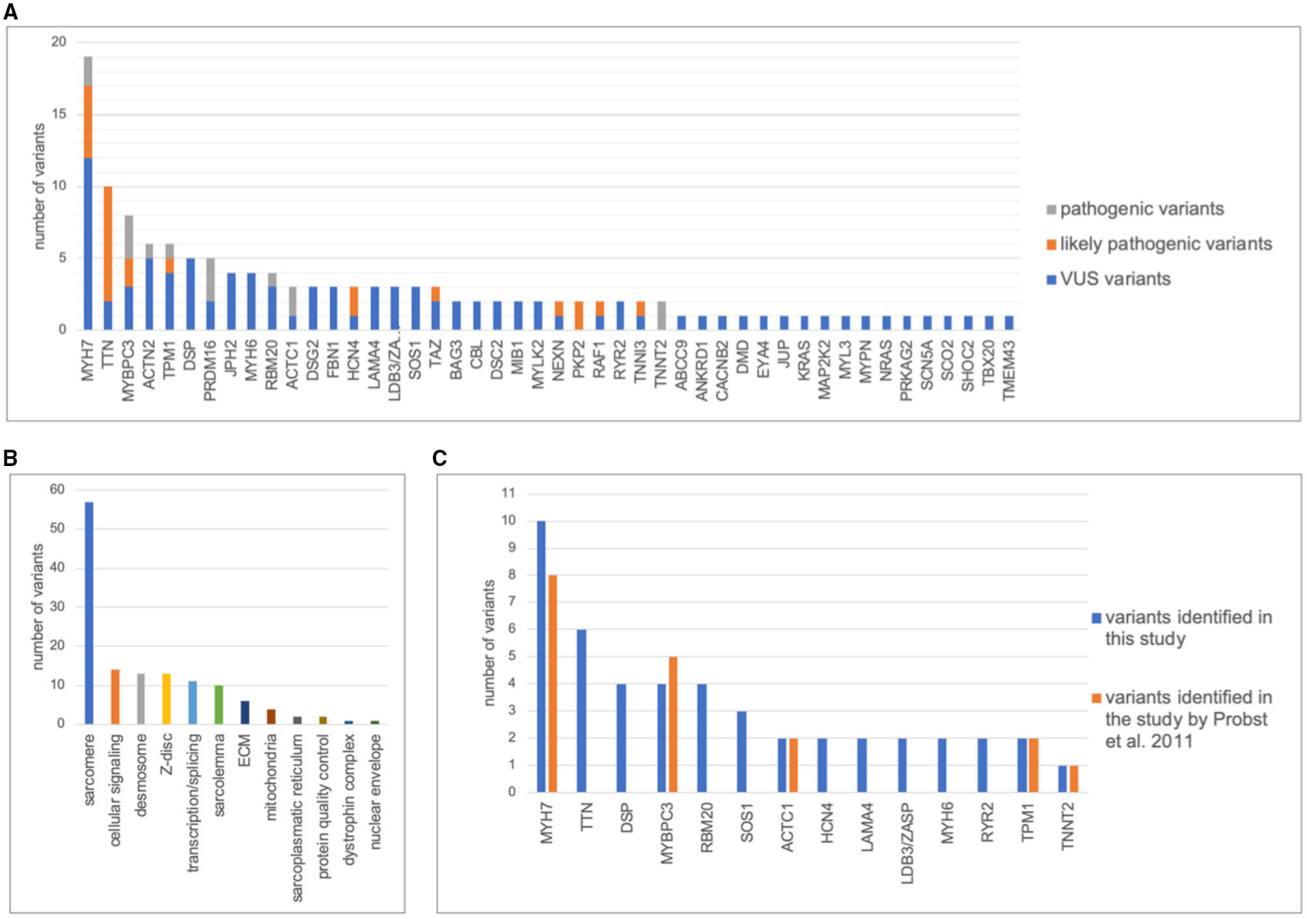
Distribution of genetic variants. Distribution of variants in cardiomyopathy genes including pathogenic, likely pathogenic, and variants of unknown significance (VUS). The figure only contains the most frequent genes. **(A)**, Number of variants detected per gene. **(B)**, Distribution of variants between functional groups. **(C)**, Number of variants found in 63 index patients by Sanger sequencing of 8 cardiomyopathy genes by Probst et al., 2011 (26) compared with next generation sequencing of 89 cardiomyopathy genes in this study.

A total of 134 variants were identified in 87/113 (77%) index patients. Ninety-three of those variants were classified as VUS, 24 as likely pathogenic and 15 as pathogenic (Table 2, Supplementary Table 1). The genetic yield of (likely) pathogenic variants was 31% corresponding to 35/113 index patients. Missense variants (*n* = 94; 70.1%) were observed most often. Variants occurred most frequently in *MYH7* (*n* = 19), *TTN* (*n* = 10) and *MYBPC3* (*n* = 8) (Figure 2A). Altogether, variants in sarcomere genes constituted 42.5% (*n* = 57) of all variants (Figure 2B). The testing of family members for the variant found in the respective index patient revealed 78 variant carriers. 124 family members did not carry variants or were not tested (Figure 1).

**Table 2:** Genetic findings in unrelated LVNC index patients.

	**All *n* = 113**
Patients with 0 variants	26 (23)
Patients with 1 variant	53 (47)
Patients with 2 variants	23 (20)
Patients with ≥3 variants	11 (10)
Patients with VUS variant	69 (61)
Patients with (likely) pathogenic variant	35 (31)
Patients with VUS only	52 (46)
Patients with (likely) pathogenic variants only	18 (16)
Patients with VUS and (likely) pathogenic variants	17 (15)
Total variants, *n*	134
Total VUS, *n*	95
Total likely pathogenic variants, *n*	24
Total pathogenic variants, *n*	15
**De novo variants**	
Yes	6
No	39
Unknown	89
**Type of variants**	
Missense, *n*	94
Frameshift, *n*	11
Stop gain, *n*	9
Splice site, *n*	17
Heterozygous variants, *n*	129
Homozygous variants, *n*	1
Hemizygous variants, *n*	4
Compound heterozygote, *n*	1

*Values are given as n (%). LVNC, Left ventricular noncompaction cardiomyopathy; VUS, Variant of uncertain significance*.

Previously published Sanger sequencing of 8 genes in 63 patients included in this study had resulted in 18 pathogenic variants in 5 genes (26). Through bioinformatic reevaluation with current ACMG guidelines 2/18 variants were not reported in this study because the MAF was > 0.0001. Through NGS, 47 additional variants were identified in 31 different genes in 43/63 patients (Figure 2C). Most additional variants were found in *TTN* which was not included in Sanger sequencing of the previous study. Of those additional 47 variants, 8 were classified as (likely) pathogenic. Altogether, we report a genetic yield of (likely) pathogenic variants in 16/63 (25%) patients in targeted panel sequencing compared to 18/63 (29%) patients in our previous study (26).

### Genetic Variants and Phenotype

Kaplan-Meier analysis did not show differences in event-free survival between the four groups classified according to presence of genetic variants. The presence of variants in specific genes did not affect event-free survival time nor was it associated with specific phenotypes (data not shown). Between patients with variants in sarcomere genes and patients without variants in sarcomere genes no differences for the risk of MACE were found (HR: 0.73; CI 95%: 0.31–1.72).

### Follow-Up

Overall, 27 events classified as MACE occurred in the study cohort during a median follow-up time of 5.6 (1.7–11.4) years. We had follow-up echocardiography data available for 89 patients. Out of those, 44% (*n* = 39) had a reduced LV systolic function at first echo and 40% (*n* = 36) at follow-up. 48% (*n* = 43) had an elevated LVEDD at first presentation and 35% (*n* = 31) at follow-up.

### Echocardiography

**Figure 3:**
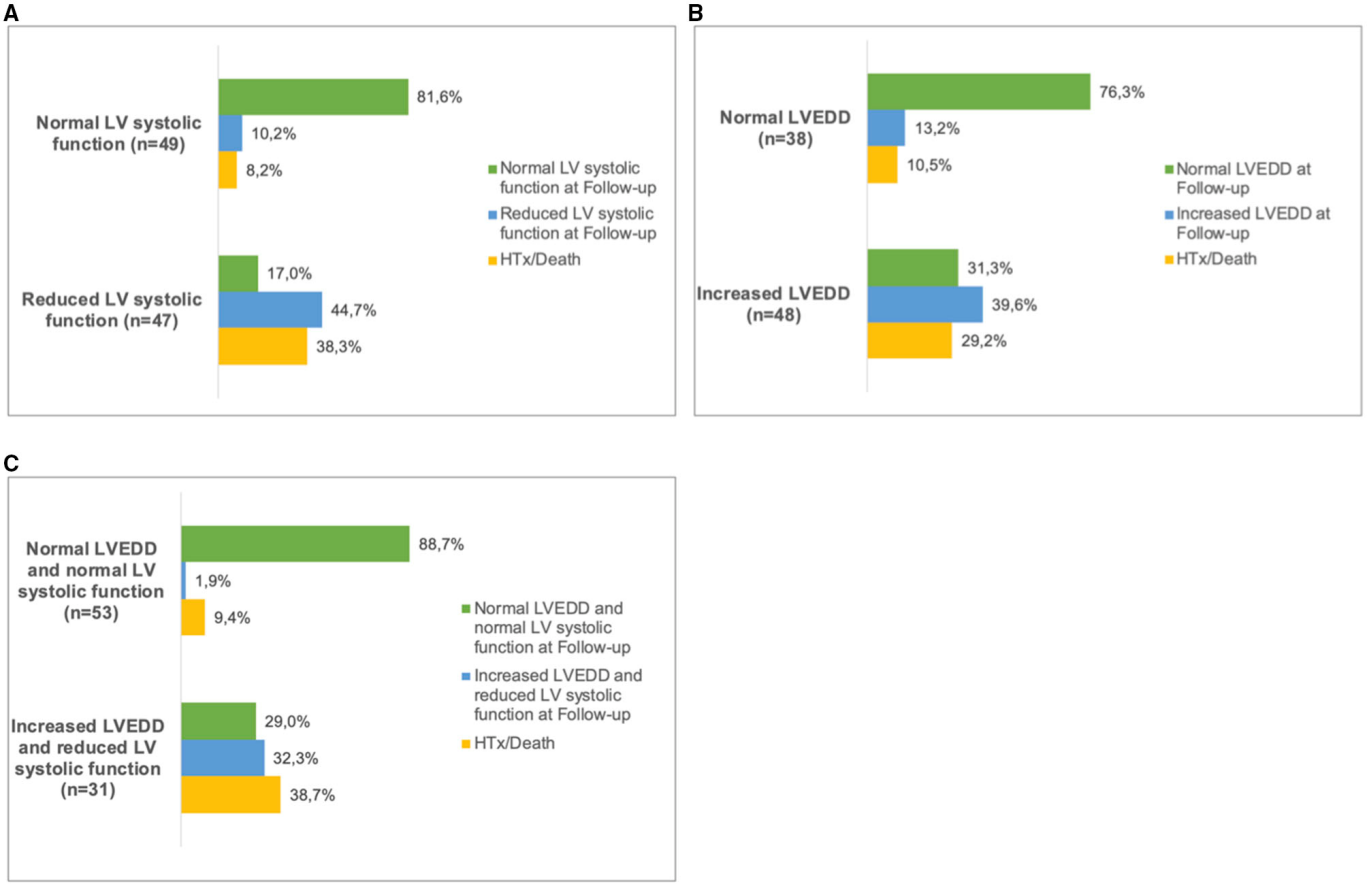
Follow up in LVNC patients. Patients were sorted into subgroups according to their phenotype at first presentation. **(A)**, normal LV systolic function and reduced LV systolic function. **(B)**, normal LVEDD and increased LVEDD and **(C)**, 'normal LVEDD and normal LV systolic function' and ‘increased LVEDD and reduced LV systolic function'. At the last available follow-up, the respective phenotypes were recorded. In some of the patients, heart transplantation (HTx) or death had occurred. LV, left ventricular; LVEDD, Left ventricular end-diastolic diameter.

**Figure 4:**
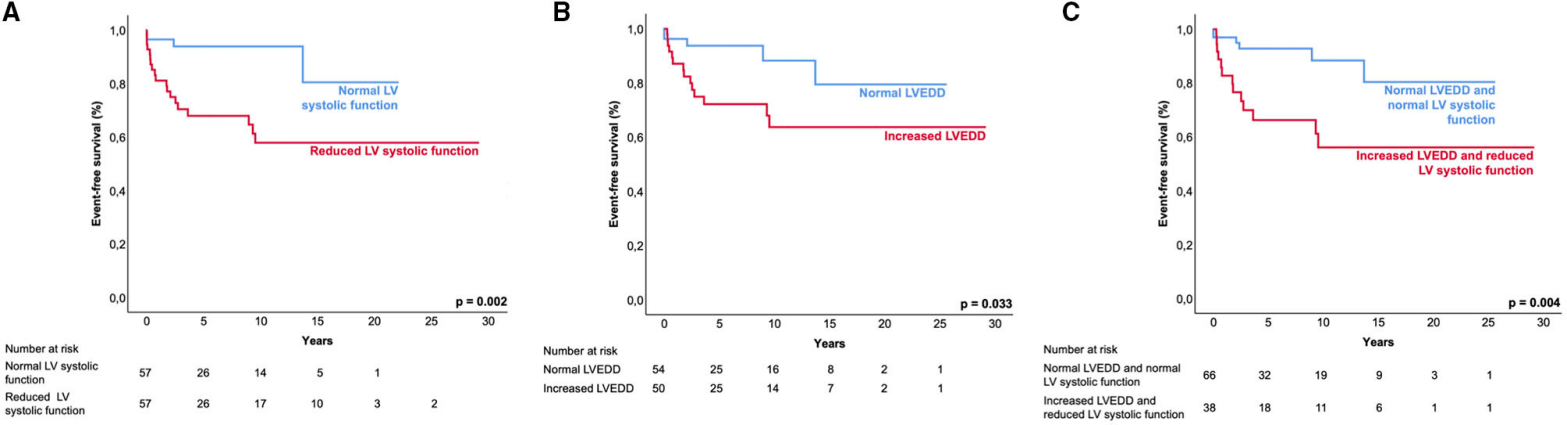
Event-free survival of LVNC patients. Kaplan-Meier analysis shows the event-free survival to the combined endpoint of mechanical circulatory support, heart transplantation, survived sudden cardiac death, and all-cause death. Event-free survival between patient groups. **(A)**, normal LV systolic function and reduced LV systolic function; **(B)**, normal LVEDD and increased LVEDD; and **(C)**, ‘normal LVEDD and normal LV systolic function' and ‘increased LVEDD and reduced LV systolic function'. LV, left ventricular; LVEDD, left ventricular end-diastolic diameter.

Patients with both, increased LVEDD and reduced LV systolic function were more often symptomatic (78 vs. 43%, *p* < 0.001) and had more ICDs implanted (33 vs. 11%, *p* = 0.003) than patients without increased LVEDD and normal LV systolic function (Table 3). 38.3 % of patients with reduced LV systolic function at first echo underwent Htx or died during follow-up, compared to only 8.2% of patients with normal LV systolic function (Figure 3A). 29.2% of patients with increased LVEDD at first echo and 38.7% with the combination of increased LVEDD and reduced LV systolic function at first presentation underwent Htx or died during follow-up (Figures 3B,C). Reduced LV systolic function, increased LVEDD, and a combination of both were risk factors for shorter event-free survival in the Kaplan-Meier analysis (Figure 4). Multivariate analysis revealed reduced LV systolic function as risk factor for event-free survival. Patients with reduced LV systolic function had 4.6 -fold higher risk for MACE (Table 4).

**Table 3:** LVNC subtypes and echocardiographic parameters.

	**SUBTYPES**					**LVEDD AND LV SYSTOLIC FUNCTION**			
	**All** ***n* = 109**	**Isolated LVNC*n* = 52(48%)**	**Dilated LVNC** ***n* = 35 (32%)**	**Hypertrophic LVNC*n* =22 (20%)**	**P-value**	**All*n* = 120**	**Normal LVEDD and normal LV systolic function** ***n* = 81 (68%)**	**Increased LVEDD and reduced LV systolic function*n* = 39 (33%)**	***P*-value**
Female	39 (36)	21 (40)	10 (29)	8 (36)	0.529	44 (37)	34 (42)	10 (26)	0.082
Age at diagnosis (yrs)	27.2 (10.4–44.7)	26.6 (17.9–42.2)	33.6 (11.6–50.0)	1.9 (0.2–29.2)	**0.029**	28.2 (10.7–44.7)	24.3 (10.4–40.0)	38.5 (11.6–52.6)	0.092
<18 years at diagnosis	40 (40)	13 (29)	10 (31)	18 (77)	** <0.001**	46 (38)	35 (43)	11 (28)	0.113
Body surface area (m^2^)	1.66 (1.15–1.92)	1.67 (1.50–1.92)	1.79 (1.53–1.99)	0.96 (0.32–1.54)	**0.001**	1.64 (1.18–1.90)	1.56 (1.09–1.86)	1.76 (1.46–1.93)	0.107
Symptomatic	54 (57)	23 (52)	22 (69)	9 (47)	0.232	61 (55)	32 (43)	29 (78)	** <0.001**
**Congenital heart defect**	21 (19)	10 (19)	3 (9)	8 (36)	**0.035**	20 (17)	17 (21)	3 (8)	0.067
Ventricular septal defect	10 (9)	4 (8)	1 (3)	5 (23)	0.055	9 (8)	9 (11)	0 (0)	**0.030**
Patent foramen ovale	8 (7)	4 (8)	1 (3)	3 (14)	0.318	10 (8)	7 (9)	3 (8)	1.000
Ebstein anomaly	3 (3)	1 (2)	1 (3)	1 (5)	0.779	3 (3)	3 (4)	0 (0)	0.550
Patent ductus arteriosus	4 (4)	2 (4)	1 (3)	1 (5)	1.000	4 (3)	3 (4)	1 (3)	1.000
Other congenital heart defects	5 (5)	3 (6)	1 (3)	1 (5)	0.851	4 (3)	4 (5)	0 (0)	0.303
**Echocardiography**									
Reduced LV systolicfunction	48 (45)	16 (31)	25 (74)	7 (35)	** <0.001**	56 (47)	17 (21)	39 (100)	** <0.001**
LV-EF (%)	48.0 (35.0–63.0)	54.0 (42.0–64.5)	37.0 (27.0–47.8)	58.5 (28.0–71.5)	**0.001**	46.8 (34.0–64.0)	57.5 (45.5–65.5)	31.0 (20.0–40.0)	** <0.001**
Increased LVEDD	43 (43)	1 (2)	32 (100)	10 (46)	** <0.001**	55 (46)	16 (20)	39 (100)	** <0.001**
LVEDD (mm)(patients >18 yrsonly)	53.0 (49.0–65.0)	50.0 (48.0–53.0)	65.0 (61.0–75.0)	49.0 (42.0–53.0)	** <0.001**	54.5 (49.0–65.0)	50.1 (48.0–54.0)	66.0 (62.0–74.0)	** <0.001**
LVEDD Z-score(patients <18 yrsonly)	1.78 (0.39–4.39)	0.05 (−0.76–0.56)	3.98 (2.35–4.56)	2.05 (0.80–4.52)	** <0.001**	1.76 (0.40–4.39)	1.04 (0.30–2.22)	5.71 (4.39–8.76)	** <0.001**
Increased LVEDDand reduced LVsystolic function	30 (31)	1 (2)	24 (75)	5 (5)	** <0.001**	-	-	-	-
**ECG**									
ST-Depression	14 (13)	7 (14)	6 (17)	1 (5)	0.419	19 (16)	10 (12)	9 (23)	0.131
T-Inversion	14 (13)	5 (10)	5 (14)	4 (18)	0.526	18 (15)	12 (15)	6 (15)	0.935
Bundle branch block	11 (14)	4 (11)	7 (28)	0 (0)	**0.037**	18 (18)	7 (10)	11 (34)	**0.004**
**Arrhythmias**	19 (17)	5 (10)	10 (29)	4 (18)	0.072	23 (19)	9 (11)	14 (36)	**0.002**
Atrial fibrillation	2 (2)	1 (2)	1 (3)	0 (0)	1.000	2 (2)	1 (1)	1 (3)	0.546
Atrioventricular blockII°/III°	1 (1)	1 (3)	0 (0)	0 (0)	1.000	1 (1)	1 (2)	0 (0)	1.000
Supraventriculartachycardia	6 (6)	0 (0)	3 (9)	3 (14)	**0.017**	8 (7)	4 (5)	4 (10)	0.435
Ventriculartachycardia	14 (13)	4 (8)	7 (20)	3 (14)	0.213	15 (13)	5 (6)	10 (26)	**0.006**
ICD	22 (20)	6 (12)	13 (37)	3 (14)	**0.010**	22 (18)	9 (11)	13 (33)	**0.003**
**Follow-up (yrs)**	6.9 (2.2–11.4)	6.1 (0.8–11.3)	7.9 (3.0–12.4)	6.3 (3.5–10.5)	0.515	6.5 (2.2–11.5)	5.5 (2.0–11.1)	8.7 (2.4–15.2)	0.146
**Complications**									
MACE	15 (14)	4 (8)	4 (11)	7 (32)	**0.026**	22 (18)	9 (11)	13 (33)	**0.003**
HTx	7 (6)	2 (4)	2 (6)	3 (14)	0.302	11 (9)	2 (3)	9 (23)	**0.001**
Death	5 (5)	1 (2)	2 (6)	2 (9)	0.282	9 (8)	3 (4)	6 (15)	0.057

*Values are given as n (%) or median (interquartile range).*

*HTx, Heart transplantation, ICD, Implantable cardioverter defibrillator, LVEDD, Left ventricular end-diastolic diameter, LV, Left ventricular, LVNC, Left ventricular noncompaction cardiomyopathy, LV-EF, Left ventricular ejection fraction, MACE, Major adverse cardiac events.*

*Values in bold indicate statistical significance*.

**Table 4:** Risk for MACE.

	**Univariate**		**Multivariate**	
	**HR (95% CI)**	***P*-value**	**HR (95% CI)**	***P*-value**
Reduced LV systolic function	4.60 (1.56–13.55)	**0.006**	4.20 (1.11–15.89)	**0.035**
LV-EF (%)	0.94 (0.92–0.97)	** <0.001**	-	-
Increased LVEDD	2.89 (1.04–8.04)	**0.042**	1.62 (0.54–4.86)	0.393
Increased LVEDD and reduced LV systolic function	3.78 (1.44–9.96)	**0.007**	-	-
Dashes (-) indicate variables that were not included in multivariate analysis.				

*LVEDD, Left ventricular end-diastolic diameter, LV, Left ventricular, LV-EF, Left ventricular ejection fraction, MACE, Major adverse cardiac events.*

*Values in bold indicate statistical significance*.

### Adult Versus Pediatric Patients

The genetic variant burden of pediatric vs. adult patients can be found in Supplementary Table 2. Adult patients were significantly more symptomatic than pediatric patients and presented with reduced LV systolic function, had more ECG-abnormalities and a higher rate of ICDs. In the pediatric cohort we found a higher prevalence of hypertrophic LVNC (Supplementary Table 3). The presence or absence of variants did not correlate with the risk of developing a MACE or the event-free survival time, neither in the pediatric nor in the adult cohort. As shown in Supplementary Table 4, hazard ratio analysis identified lower BSA, lower LV-EF (%), increased LVEDD and the presence of symptoms as factors for a higher risk for MACE in our cohort. In adults, an older age at diagnosis increased the risk for MACE. In pediatric patients, age at diagnosis had no impact on MACE. Multivariate analysis revealed lower LV-EF as independent risk factor for MACE in the whole cohort and in the pediatric subcohort (Supplementary Table 5).

### Pediatric Patients

In pediatric patients, a lower BSA of 0.1 m^2^ increased the risk of MACE by 7.4%. The LV-EF reduction was the main risk factor with a higher independent impact than a lower BSA or increased LVEDD. The risk for MACE was decreased by approximately 8% for each additional percent of LV-EF (for comparison 4% in adults) (Supplementary Tables 4, 5).

### Phenotypic Subtypes

We classified 109 patients into the LVNC CMP subtypes. 52 (47.7%) presented with isolated LNVC CMP, 35 (32.1%) with dilated LVNC CMP and 22 (20.2%) with hypertrophic LVNC CMP (Table 3). Patients with hypertrophic LVNC CMP were younger at diagnosis, more frequently affected by congenital heart defects, and at higher risk (OR: 4.61; CI 95%: 1.45–14.63) for MACE (*p* = 0.01). Patients with dilated LVNC presented more frequently with a reduced LV systolic function, had the highest rate of arrhythmias (31%), and ICDs implanted (37%). The presence of dilated LVNC CMP did not have an impact on the likelihood of MACE (OR: 0.74; CI 95%: 0.22–2.51) despite a lower LV-EF, neither did the presence of isolated LVNC (OR: 0.35; CI 95%: 0.10–1.17). The analysis of event-free survival did not show any differences between the subtypes as shown in Supplementary Figure 1.

### Genetic Characteristics and Clinical Outcome

The presence or absence of (likely) pathogenic variants or variants in specific genes did not allow a risk stratification for MACE or the duration of event-free survival (data not shown). The presence of one or multiple VUS variants in addition to a (likely) pathogenic variant in a patient also failed to correlate with a higher risk for MACE than only a (likely) pathogenic variant (HR: 2.17; CI 95%: 0.40–11.90).

## Discussion

We investigated a cohort of 113 pediatric and adult LVNC CMP patients for genetic and clinical parameters to predict outcome. We included affected and unaffected family members from 54 families. We identified reduced LV systolic function as a strong, independent risk factor for MACE. In pediatric patients, a lower BSA and lower LV-EF predicted a worse outcome. Genetic variants did not correlate with clinical outcome. Altogether, the genetic yield of (likely) pathogenic variants using targeted panel sequencing was 31%, well comparable to previous studies. Genetic screening should be focused on validated genes and is useful in family counseling.

### Implications for Outcome

Echocardiography is used most commonly for diagnosis according to the Jenni criteria (1), and also seems to be the best, widely available tool for basic risk stratification.

Adult LVNC CMP patients with normal LV function were reported to have no higher mortality than the general population (8). Multivariate analysis identified age at diagnosis and LV dilatation as independent risk factors (8). Left ventricular dilation and systolic dysfunction were less strong predictors for survival than New York Heart Association class III/IV and cardiovascular complications at presentation (27). According to our results, reduced LV systolic function is the most important prognostic factor for clinical outcome (28, 29). Asymptomatic patients with normal echocardiography mostly remain with normal cardiac function during long-term follow-up. There is a clear association between symptomatic patients with abnormal echocardiographic findings and an impaired long-term clinical outcome. Previous reports described a noncompaction phenotype in pregnancy, athletes and other cardiac healthy individuals without functional impairment (30–32). In these patients, noncompaction is often reversible, does not affect cardiac function and is not associated with a CMP. Therefore, LVNC should not be labeled as a cardiomyopathy under these circumstances. The judgement of the phenotype as a disease should therefore probably rather be made by functional parameters determined by echocardiography or CMR imaging (33). In an adult cohort an association between reduced LV systolic function and mid-basal wall involvement was shown (8). Deeper phenotyping by CMR imaging showed that diffuse myocardial fibrosis contributed to heart failure in a pediatric DCM cohort and may lead to new clues in pediatric LVNC, as well (34).

### Adult Versus Pediatric Patients

A systematic review of a larger LVNC cohort reported on worse clinical outcome in children (35). This was not found in our cohort and may be due to an older range of the pediatric cohort (median age 1.9 vs. 0 years) and exclusion of children with genetic syndromes, chromosomal defects, and neuromuscular symptoms. Nevertheless, lower BSA and younger age are considered risk factors for MACE. Our study showed a higher rate of asymptomatic children compared to asymptomatic adults, which might be explained by a referral bias of asymptomatic adults being sent less frequently to tertiary centers of this study. The rate of 31% asymptomatic adults was comparable to other adult cohorts (8).

### The Impact of LVNC Subtypes

Based on the classification by Van Waning et al. we used 3 subgroups to classify our patients (18). Nearly half of the cohort in their study (18) and in this study were classified as isolated LVNC CMP without dilatation or hypertrophy, 42 and 48%, respectively. These findings support the general consensus defining LVNC as a distinct myocardial disease. In some cases, an overlap with other cardiomyopathies might still be suspected, especially because family members with DCM or HCM without noncompaction can also be found (18, 36). Additionally, many of the mutated genes were described causing other primary cardiomyopathies (14, 15). Meanwhile, LVNC-specific variants probably explain 5–10% of cases (16). It has been shown, that pediatric patients with isolated LVNC CMP have the best outcome compared to patients with LVNC and an underlying DCM/HCM (5). One might suspect an overlap with noncompaction without cardiomyopathy, like it has been discussed before (33).

### Genotype-Phenotype Correlation

Mutations in *MYH7, TTN* and *MYBPC3* were most prevalent in our study, as described by others (35, 37). The evidence for genotype-phenotype correlations remains controversial (4, 38). Nevertheless, with the focus on an impaired LV systolic function of pediatric patients with LVNC CMP, van Waning et al. suggest that including genetic findings in children may be helpful predicting clinical outcome and may be appropriate in clinical management (4). On the other hand, genetic counseling is recommended, for young patients and valuable for family counseling (35).

Variants in specific genes were associated with worse outcome in LVNC, as reported for variants in *Lamin A/C, RBM20, TAZ*, Titin-truncating variants and non-sarcomere genes in general (13, 37, 39, 40). Overall, larger cohorts, and genotype-phenotype studies analyzing the correlation between genetic background and clinical outcome are needed in the future. Based on these findings more patient-individual genetic counseling and more precise disease management becomes possible.

### Family Screening

Potential non-penetrance of variants, as described in systematic family screening of pediatric primary cardiomyopathies before (41), might be a reason for asymptomatic variant carriers identified through family screening. One possible explanation for intra-familial variability might be the role of genetic modifiers.

### Limitations

Our cohort was heterogenous and consisted of patients from different hospitals. Clinical data was collected from medical reports from different physicians and an information bias cannot be ruled out. Also, genetic and clinical data on family members was not available for many index patients. Clinical data from adults and children cannot always be directly compared. Therefore, we converted numerical data into dichotomous variables. Our limited cohort size led to small subgroups, which limited the statistical power. Especially in the pediatric cohort, syndromic comorbidities and other heart defects may not always be identified or reported. A referral bias of more severe cases is possible.

## Conclusions

We performed a retrospective study on a large cohort of LVNC CMP patients to determine genetic and clinical factors to predict the clinical course and outcome of LVNC. We report that reduced LV systolic function is a risk factor for MACE in pediatric patients and in adults. The presence or absence of genetic variants was not predictive for the risk of developing a MACE, neither in the pediatric nor in the adult cohort. Genetic screening is useful for cascade screening to identify family members at risk for developing LVNC CMP.

## Data Availability Statement

The original contributions presented in the study are included in the article/Supplementary Material, further inquiries can be directed to the corresponding author/s.

## Ethics Statement

The studies involving human participants were reviewed and approved by Charite Universitätsmedizin Berlin, Berlin, Germany. Written informed consent to participate in this study was provided by the participants' legal guardian/next of kin.

## Author Contributions

AS-B and SK contributed to conception, design of the study, and wrote the first draft of the manuscript. WK, RJ, MG, EO, and FB contributed patient data. AS-B, JK, CH, FS, NA-W-M, JD, and ST analyzed clinical and genetic data and organized the database. AS-B performed the statistical analysis. All authors contributed to manuscript revision, read, and approved the submitted version.

## Conflict of Interest

The authors declare that the research was conducted in the absence of any commercial or financial relationships that could be construed as a potential conflict of interest.

## Publisher's Note

All claims expressed in this article are solely those of the authors and do not necessarily represent those of their affiliated organizations, or those of the publisher, the editors and the reviewers. Any product that may be evaluated in this article, or claim that may be made by its manufacturer, is not guaranteed or endorsed by the publisher.

## Abbreviations

BSA: Body surface area
CMR: Cardiac magnetic resonance
DCM: Dilated cardiomyopathy
HCM: Hypertrophic cardiomyopathy
ICD: Implantable cardioverter defibrillator
LVEDD: Left ventricular end-diastolic diameter
LV: Left ventricular
LVNC: Left ventricular noncompaction cardiomyopathy
LV-EF: Left ventricular ejection fraction
MACE: Major adverse cardiac events
NGS: Next-generation sequencing
VUS: Variant of uncertain significance.
