# Supplementary material for: Reduced Systolic Function and Not Genetic Variants Determine Outcome in Pediatric and Adult Left Ventricular Noncompaction Cardiomyopathy
Source: Front Pediatr. 2021 Sep 3;9:722926. doi: 10.3389/fped.2021.722926 (PMC8447880; doi:10.3389/fped.2021.722926)
Supplement: Supplementary file 1 [file Data_Sheet_1.PDF]

Supplemental Material

Supplemental Table 1. Pathogenic and likely pathogenic variants in 113 unrelated index patients with LVNC

Gene	Transcript	cDNA alteration	Protein alteration	gnomAD allele frequency	de novo	Pathogenicity	ACMG terms
ACTC1*	NM_005159.4	c.301G>A	p.E101K	4.061E-06	no	P	PS1, PS3, PS4
ACTC1*	NM_005159.4	c.301G>A	p.E101K	4.061E-06	no	P	PS1, PS3, PS4
ACTN2	NM_001103.2	c.574C>T	p.R192*	8.122E-06	yes	P	PM2, PM6, PVS1
HCN4	NM_005477.2	c.1445G>A	p.G482E	0	no	LP	PM2, PM5, PS3
HCN4	NM_005477.2	c.1454C>T	p.A485V	7.217E-06	?	LP	PP3, PM2, PS3
MYBPC3	NM_000256.3	c.709T>C	p.Y237H	0	?	P	PM2, PS1, PS3
MYBPC3*	NM_000256.3	c.1484G>A	p.R495Q	0.00002031	?	P	PS1, PS3, PS4
MYBPC3	ENSG00000134571	c.1805C>T	p.T602I	9.848E-06	no	LP	PM2, PS1
MYBPC3	NM_000256.3	c.2572A>C	p.S858R	0	yes	LP	PM2, PM5, PS2
MYBPC3*	NM_000256.3	c.2864-2865delCT	p.P955Rfs*95	0.00003232	?	P	PS4, PVS1
MYH7*	NM_000257.2	c.715-717GACdel	p.D239-	0	?	LP	PM1, PM2, PS1
MYH7*	NM_000257.2	c.728G>A	p.R243H	8.121E-06	no	P	PP1, PM1, PS1, PS4
MYH7*	NM_000257.2	c.754T>C	p.F252L	0	?	LP	PM1, PM2, PM5
MYH7	NM_000257.2	c.847T>G	p.Y283D	0	no	LP	PP3, PM1, PS4
MYH7	NM_000257.2	c.1048T>A	p.Y350N	0	yes	LP	PP3, PM1, PM2, PM6
MYH7	ENSG00000092054	c.1283C>A	p.A428D	0	no	LP	PP1, PP3, PM1, PM2
MYH7	NM_000257.2	c.2770G>A	p.E924K	0	no	P	PM1, PS1, PS4
NEXN	NM_144573.3	c.1876-1877Adel	p.E626EX	0	?	LP	PM2, PM4, PS3
PKP2	NM_004572.3	c.1069-1070AGdel	p.V357Efs*29	0	?	LP	PM1, PM2, PM4
PKP2	NM_004572.3	c.2393-2401CATTGAACAdel	p.TLNN798-800N	0	?	LP	PM1, PM2, PM4
PRDM16	NM_022114.3	c.1573Cdel	p.R525Pfs*79	0	yes	P	PS3, PS4, PVS1

PRDM16	NM_022114.3	c.1627C>T	p.Q543*	0	?	P	PM2, PS3, PVS1
PRDM16	NM_022114.3	c.2104A>T	p.K702*	0	yes	P	PS3, PS4, PVS1
RAF1	NM_002880.3	c.806C>T	p.T269I	0	?	LP	PP3, PM2, PS3
RBM20	NM_001134363.1	c.2737G>A	p.E913K	0	no	P	PS1, PS3, PS4
TAZ	NM_000116.3	c.355G>A	p.V119M	0	no	LP	PM1, PM2, PM5
TNNI3	NM_000363.4	c.428C>A	p.T143N	0.00004071	?	LP	PM1, PS4
TNNT2*	NM_000364.2	c.421C>T	p.R141W	0	yes	P	PS1, PS3, PS4
TNNT2	NM_001276345.1	c.460C>T	p.R154W	0.00003676	?	P	PM2, PS1, PS3
TPM1	NM_001018005.1	c.257C>T	p.A86V	0	no	LP	PP1, PP3, PM1, PM2
TPM1*	NM_001018004.1	c.574G>A	p.E192K	0	?	P	PS1, PS4
TTN	NM_001267550.1	c.4714C>T	p.R1572*	0	no	LP	PM1, PM2, PM4
TTN	NM_001267550.1	c.4724_4728delTG AAA	p.M1575Sfs*6	0.00001084	?	LP	PM1, PM2, PM4
TTN	NM_001267550.1	c.63601C>T	p.R21201*	0.00001089	?	LP	PM1, PM2, PS1
TTN	NM_001267550.1	c.63601C>T	p.R21201*	0.00001089	?	LP	PM1, PM2, PS1
TTN	NM_001267550.1	c.70879C>T	p.Q23627*	0	?	LP	PM1, PM2, PM4
TTN	NM_001267550.1	103360	p.E34454Nfs*3	0.0000217	?	LP	PM1, PM2, PM4
TTN	NM_001267550.1	c.103360Gdel	p.E34454X	0.0000217	?	LP	PM1, PM2, PS1
TTN	NM_001267550.1	c.107284C>T	p.R35762*	0	?	LP	PM1, PM2, PM4

*Previously published variants (Sanger sequencing of 8 genes) in Probst et al. (PMID: 21551322).
LP = Likely pathogenic; P = Pathogenic; VUS = Variant of uncertain significance

Supplemental Table 2. Genetic findings in pediatric and adult patients

	All n=109	<18 years at diagnosis n=43	>18 years at diagnosis n=66
Patients with 0 variants	25 (23)	11 (26)	14 (21)
Patients with 1 variant	51 (47)	19 (44)	32 (49)
Patients with 2 variants	22 (20)	10 (23)	12 (18)
Patients with ≥3 variants	11 (10)	3 (7)	8 (12)
Patients with VUS only	49 (45)	20 (47)	29 (44)
Patients with (likely) pathogenic variant only	18 (17)	5 (12)	13 (20)
Patients with VUS and (likely) pathogenic variants	17 (16)	7 (16)	10 (15)
Total variants, n	130	48	82
Total VUS, n	92	34	58
Total likely pathogenic variants, n	24	9	15
Total pathogenic variants, n	14	5	9

Values are given as n (%).

Abbreviations: VUS = Variant of uncertain significance

Supplemental Table 3. Clinical characteristics in pediatric and adult patients

	All n=137	<18 years at diagnosis n=55 (40%)	>18 years at diagnosis n=82 (60%)	P-Value
Female	54 (39)	26 (47)	28 (34)	0.123
Age at diagnosis (yrs)	27.8 (9.2-44.7)	1.9 (0.2-10.7)	40.3 (29.0-54.1)	**<0.001**
Body surface area (m^2)	1.64 (1.15-1.89)	0.95 (0.33-1.43)	1.81 (1.63-1.96)	**<0.001**
Symptomatic	68 (55)	17 (34)	51 (69)	**<0.001**
**Congenital heart defect**	23 (17)	13 (24)	10 (12)	0.079
Ventricular septal defect	11 (8)	8 (15)	3 (4)	**0.027**
Patent foramen ovale	10 (7)	7 (13)	3 (4)	0.089
Ebstein anomaly	5 (4)	2 (4)	3 (4)	1.000
Patent ductus arteriosus	4 (3)	4 (7)	0 (0)	**0.024**
Other congenital heart defects	4 (3)	1 (2)	3 (4)	0.649
**Echocardiography**				
Reduced LV systolic function	61 (46)	17 (33)	44 (55)	**0.012**
LV-EF (%)	46.8 (33.0-64.0)	57.0 (44.0-67.0)	43.0 (33.0-55.0)	**0.001**
Increased LVEDD	55 (45)	21 (45)	34 (45)	0.944
LVEDD (mm)	50.0 (42.0-60.0)	39.0 (30.0-48.0)	54.0 (49.0-65.0)	**<0.001**
LVEDD (Z-score)		1.66 (0.40-4.39)		
Increased LVEDD and reduced LV systolic function	39 (33)	11 (24)	28 (38)	0.113
**Subtypes**				
LVNC	45 (46)	13 (33)	32 (54)	**<0.001**
Dilated LVNC	32 (32)	10 (25)	22 (37)	
Hypertrophic LVNC	22 (22)	17 (43)	5 (9)	
**ECG**				
ST-Depression	20 (15)	3 (5)	17 (21)	**0.013**
T-Inversion	22 (16)	5 (9)	17 (21)	0.069
Bundle branch block	21 (19)	2 (5)	19 (28)	**0.002**
Arrhythmias	25 (18)	5 (9)	20 (24)	**0.007**
Atrial fibrillation	2 (2)	0 (0)	2 (2)	0.516
Atrioventricular block II°/III°	1 (1)	1 (2)	0 (0)	1.000

Supraventricular tachycardia	8 (6)	2 (4)	6 (7)	0.475
Ventricular tachycardia	17 (12)	4 (7)	13 (16)	0.135
ICD	24 (18)	2 (4)	22 (27)	**<0.001**
Follow-up (yrs)	5.6 (1.8-11.4)	3.5 (1.5-7.4)	7.8 (1.8-13.7)	**0.016**
**Complications**				
MACE	27 (20)	12 (22)	15 (18)	0.611
HTx	14 (10)	9 (16)	5 (6)	0.052
Death	11 (8)	2 (4)	9 (11)	0.121

Values are given as n (%) or median (interquartile range).

Abbreviations: HTx = Heart transplantation, ICD = Implantable cardioverter defibrillator, LVEDD = Left ventricular end-diastolic diameter, LV = Left ventricular, LVNC = Left ventricular noncompaction cardiomyopathy, LV-EF = Left ventricular ejection fraction, MACE = Major adverse cardiac events

Supplemental Table 4

Hazard ratio – Risk for MACE – univariate

	all		<18 years at diagnosis		>18 years at diagnosis	
	**HR (95% CI)**	**P-Value**	**HR (95% CI)**	**P-Value**	**HR (95% CI)**	**P-Value**
Age at diagnosis (yrs)	1.00 (0.98-1.02)	0.825	0.81 (0.64-1.03)	0.082	1.04 (1.01-1.08)	**0.019**
Body surface area (m^2)	0.51 (0.27-0.97)	**0.039**	0.16 (0.03-0.97)	**0.047**	0.41 (0.05-3.33)	0.404
LV-EF (%)	0.94 (0.92-0.97)	**<0.001**	0.92 (0.87-0.96)	**0.001**	0.96 (0.93-1.00)	**0.036**
Increased LVEDD	2.89 (1.04-8.04)	**0.042**	3.97 (0.46-34.70)	0.212	2.88 (0.90-9.20)	0.074
LVEDD (mm)	-	-	-	-	1.05 (1.00-1.10)	**0.032**
LVEDD (Z-score)	-	-	1.50 (1.14-1.98)	**0.004**	-	**-**
Increased LVEDD and reduced LV systolic function	3.78 (1.44-9.96)	**0.007**	11.97 (1.39-103.22)	**0.024**	2.82)0.94-8.43)	0.064
Symptomatic	4.83 (1.43-16.33)	**0.011**	3.40 (0.79-14.59)	0.099	6.46 (0.85-49.21)	0.072
Arrhythmias	2.03 (0.86-4.79)	0.108	1.37 (0.25-7.50)	0.718	2.19 (0.78-6.19)	0.138

Abbreviations: LVEDD = Left ventricular end-diastolic diameter, LV = Left ventricular, LV-EF = Left ventricular ejection fraction, MACE = Major adverse cardiac events

Supplemental Table 5

Hazard ratio – Risk for MACE – multivariate

	all		<18 years at diagnosis		>18 years at diagnosis	
	**HR (95% CI)**	**P-Value**	**HR (95% CI)**	**P-Value**	**HR (95% CI)**	**P-Value**
Age at diagnosis (yrs)	-	-	-	-	1.04 (0.996-1.08)	0.076
Body surface area (m^2)	0.36 (0.15-0.88)	**0.026**	0.03 (0.00-2.95)	0.130	-	-
LV-EF (%)	0.94 (0.91-0.98)	**0.003**	0.92 (0.86-0.99)	**0.032**	0.96 (0.91-1.02)	0.165
Increased LVEDD	0.75 (0.22-2.52)	0.643	-	-	-	-
LVEDD (mm)	-	-	-	-	0.996 (0.93-1.06)	0.914
LVEDD (Z-score)	-	-	1.37 (0.90-2.09)	0.148	-	-
Symptomatic	3.03 (0.71-12.88)	0.133	-	-	-	-
Dashes (-) indicate variables that were not included in multivariate analysis.						

Abbreviations: LVEDD = Left ventricular end-diastolic diameter, LV-EF = Left ventricular ejection fraction, MACE = Major adverse cardiac events

Supplemental Figure 1. Event-free survival between LVNC subtypes

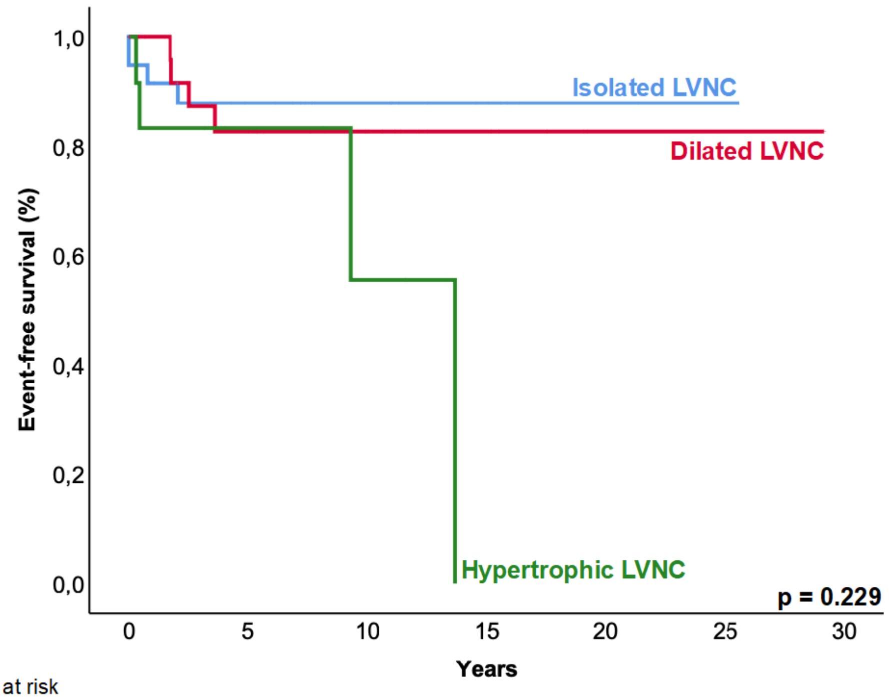

Number at risk						
Isolated LVNC	39	17	11	6	2	1
Dilated LVNC	29	18	12	6	1	1
Hypertrophic LVNC	13	5	2			

Kaplan-Meier analysis for event-free survival time between the LVNC subtypes. The combined endpoint are major adverse cardiac events (mechanical circulatory support, heart transplantation, survived sudden cardiac death and/or all-cause death).
